# Impact of gantenerumab amyloid removal therapy on established Alzheimer Disease fluid biomarkers

**DOI:** 10.1002/alz.094831

**Published:** 2025-01-09

**Authors:** Laura Ibanez, Yan Li, Eric McDade, Jorge J. Llibre‐Guerra, David B. Clifford, Susan Mills, Anna Santacruz, Guoqiao Wang, Charlene Supnet‐Bell, Tammie L.S. Benzinger, Brian A. Gordon, Gregory Klein, Monika Baudler, Rachelle S. Doody, Geoffrey A. Kerchner, Tobias Bittner, Alireza Atri, Randall J. Bateman

**Affiliations:** ^1^ Washington University in St. Louis, School of Medicine, St. Louis, MO USA; ^2^ Knight Alzheimer Disease Research Center, St. Louis, MO USA; ^3^ Knight Alzheimer Disease Research Center, St Louis, MO USA; ^4^ Washington University in St. Louis School of Medicine, St. Louis, MO USA; ^5^ Washington University School of Medicine in St. Louis, St. Louis, MO USA; ^6^ Washington University in St. Louis, St. Louis, MO USA; ^7^ F. Hoffmann‐La Roche Ltd, Basel Switzerland; ^8^ Banner Alzheimer’s Institute, Phoenix, AZ USA

## Abstract

**Background:**

Anti‐amyloid monoclonal antibody therapies have successfully removed amyloid plaque as measured using imaging techniques. However, the characteristic fluid biomarker trajectories following plaque removal remains understudied, particularly during the preclinical phases of disease. We investigated biomarker trajectories in the context of the gantenerumab open label extension (OLE) of the Dominantly Inherited Alzheimer’s Network Trials Unit (DIAN‐TU) secondary prevention trial (DIAN‐TU‐001) in Autosomal Dominant Alzheimer’s disease (ADAD) mutation carriers.

**Method:**

Cerebrospinal fluid (CSF) amyloid beta 1‐42/40 (ABeta 42/40) ratio, total tau, phosphorylated Tau 217 (pTau217) and microtubule‐binding region of tau containing residue 243 (MTBR‐Tau243) were measured using Immuno‐Precipitation followed by Mass Spectrometry (IP‐MS) in all participants. Linear mixed‐effects models assessed temporal changes in biomarker levels. Correlations between biomarker measurements, clinical data, and imaging parameters were evaluated, considering estimated age at onset (EYO), and dosage.

**Result:**

During both the DIAN‐TU‐001 double‐blind and OLE phases, we observed a significant increase in the CSF ABeta 42/40 ratio, notably among presymptomatic individuals. This change approached the levels considered to be healthy (11.58%), even in participants beyond their EYO. In addition, CSF pTau 217 levels declined in the participants treated with gantenerumab compared to the placebo group during the double blind and OLE phases in both asymptomatic and symptomatic groups. Higher doses were associated with faster rates of change for both biomarkers. A significant decrease in total tau was observed across treatment groups (gantenerumab and placebo) during the OLE phase. No changes were observed for MTBR243.

**Conclusion:**

Treatment with amyloid‐removing therapy induced changes in biomarker measurements consistent with a healthier state. Improvement in biomarker levels in individuals receiving treatment prior to their EYO supports the potential of early amyloid‐removal therapy to mitigate the risk of progression. Importantly, the rate of biomarker change was directly proportional to dosage, suggesting potential dose‐dependent benefits. Additional trials are warranted to elucidate the progression of both biomarkers and clinical symptoms after effective brain amyloid removal.

